# Clinical research and trials in echocardiography: rationale, requirements and future opportunities

**DOI:** 10.1186/s44156-025-00083-2

**Published:** 2025-08-11

**Authors:** Casey L. Johnson, Ross Upton, Samuel Krasner, Sadie Bennett, Ashley Ackerman, Xing Liu, Daniel X. Augustine, Maria F. Paton, Paul Leeson

**Affiliations:** 1 Oxford Cardiovascular Clinical Research Facility, Division of Cardiovascular Medicine, Radcliffe Department of Medicine, John Radcliffe Hospital, University of Oxford, Level 1, Oxford, OX3 9DU UK; 2 Ultromics Ltd., Oxford, UK; 3 University Hospitals of North Midlands NHS Trust, Stoke-On-Trent, UK; 4 Royal United Hospitals Bath NHS Foundation Trust, Bath, UK; 5 Leeds Institute of Cardiovascular and Metabolic Medicine, University of Leeds, Leeds, UK; 6 Leeds Teaching Hospitals Trust, Leeds, UK

**Keywords:** Echocardiography, Clinical research, Cardiovascular disease, Artificial intelligence, Clinical trials

## Abstract

Echocardiography has established itself as a vital component in the diagnosis and management of cardiovascular disease, evolving alongside advancements in imaging technology and clinical research methodologies. Since its inception in the 1950s, echocardiographic research has progressed from small-scale, observational studies to large cohort investigations and randomised controlled trials. This evolution has paralleled advancements in disease diagnosis and facilitated the use of echocardiography as an important player in other disciplines such as cardio-oncology and interventional cardiology. Echocardiography research has made great progress, with new developments rapidly shaping the field. This continued innovation underscores the singular focus of improving patient care. As digital and technological advancements accelerate, the potential for research in echocardiography to enhance diagnostic precision, guide personalised treatment, and improve outcomes on a global scale is greater than ever. Collaborative efforts and sustained investment in research will be key to realising these goals and advancing the care of patients with cardiovascular disease. This review explores the historical and ongoing contributions of echocardiography research to better understanding cardiac disease, emphasising the pivotal roles of early feasibility studies and large-scale trials in refining techniques and establishing clinical utility. Key infrastructure requirements for advancing echocardiography research are identified, including workforce development, academic and healthcare collaborations, clinical trial support, and access to big data and computational expertise. Emerging technologies, such as advanced imaging techniques, handheld devices, and AI-driven analytics, are highlighted as transformative tools poised to address current limitations in clinical practice.

## Introduction

Echocardiography plays a vital role in the diagnostic work-up of most cardiovascular conditions ranging from hypertension to valvular heart disease, coronary artery disease, heart failure, and cardiomyopathies [1]. Clinical scenarios in which echocardiography is appropriate are also increasing, covering emergency settings to non-urgent initial assessments of patients [2]. In 2023, there were around 1.6 million cardiac echocardiograms performed in England [3]. Therefore, there is great potential for echocardiography to be a key driver in our efforts to improve quality of cardiovascular care, through both evidence-based research and practice. The European Society of Cardiology has highlighted that cardiovascular disease research receives less funding than other clinical areas [4] but also identified key domains in need of additional strategic research funding to include earlier recognition of cardiovascular disease, and, in particular, non-invasive imaging in this area [5]. In this review, we highlight the key types of research studies that are performed within echocardiography, identify infrastructure requirements to support further advances in echocardiography research, and describe future opportunities in echocardiography research. We will also describe the preparedness of the UK to contribute to the advancement of knowledge in the field as novel technologies in echocardiography emerge.

## Clinical studies in echocardiography

### Clinical and technical research and development

**Fig. 1:**
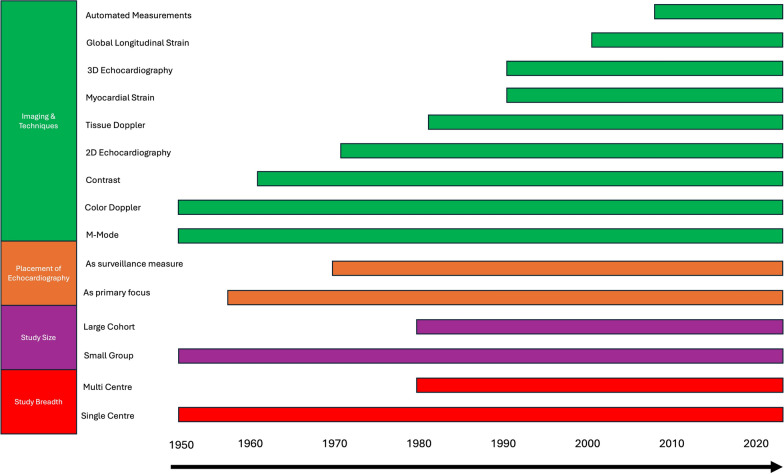
Key developments and design of transthoracic echocardiography research through time

Research has been a fundamental part of echocardiography practice since the development of the earliest echocardiographic techniques in the 1950s [6]. Early studies focused on describing the basic principles of ultrasound-based imaging, and its use as a diagnostic tool and were pivotal in establishing echocardiography in clinical practice. The first applications for using ultrasound to assess cardiac structure were described by Edler and Hertz in 1954 who investigated the use of M-mode echocardiography to illustrate heart motion [7]. During this early developmental stage of echocardiography, research was primarily designed to assess the feasibility of using ultrasound to visualise heart structures. As such, early work was purely observational in nature, often consisting of small groups of patients from single centres, individual case studies, or even collections of excised hearts [7–9]. The 1950s also saw the development of Doppler echocardiography by Satomura et al., who investigated its clinical applications across various cardiac diseases [18, 19], followed by developments in contrast echocardiography [10] and pulsed wave Doppler during the 1960s [11–13]. The first clinical research reports using 2D echocardiography were presented in 1973 by Roelandt and Kloster, along with Bom et al. [14, 15] which saw the start of larger investigations of over 100 participants. Wide availability of 2D echocardiography supported developmental work in the 1980s and 1990s in diastolic function assessment [16], tissue Doppler imaging [17] and myocardial strain [18]. While computing advances in the early 2000s led to the first commercial system for speckle tracking and the development of global longitudinal strain [19] (Fig. 1).

**Fig. 2:**
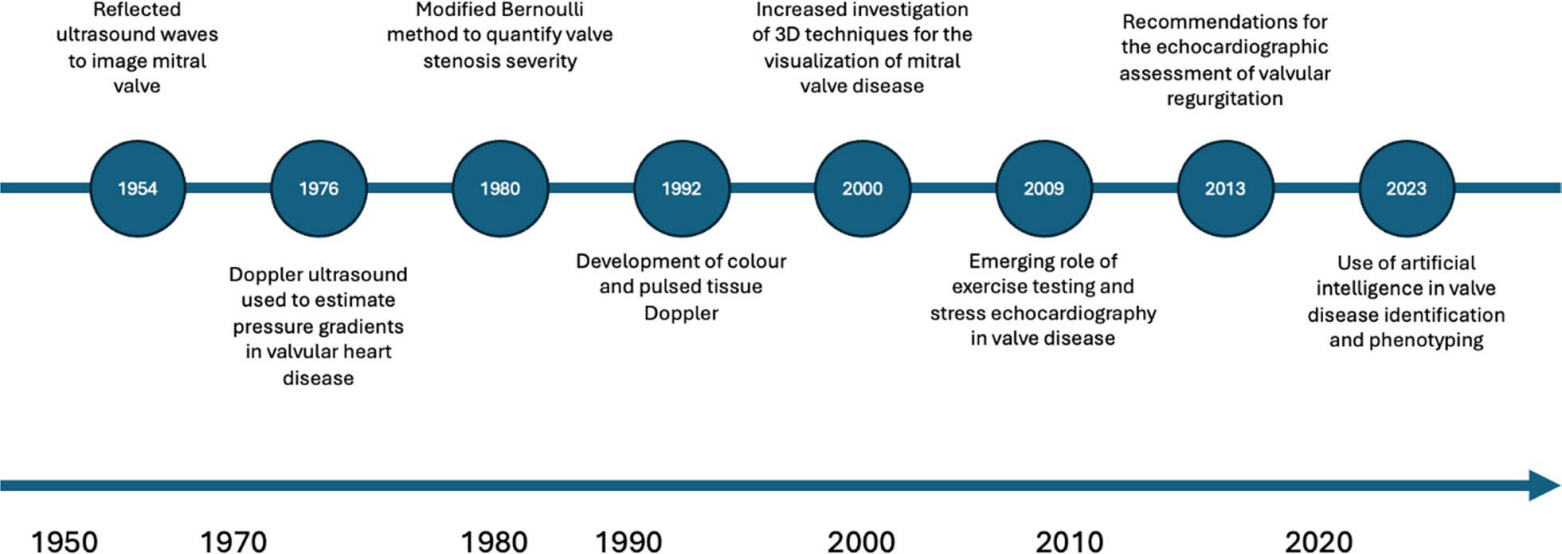
Timeline of echocardiography advancements in the identification and diagnosis of valvular heart disease

**Fig. 3:**
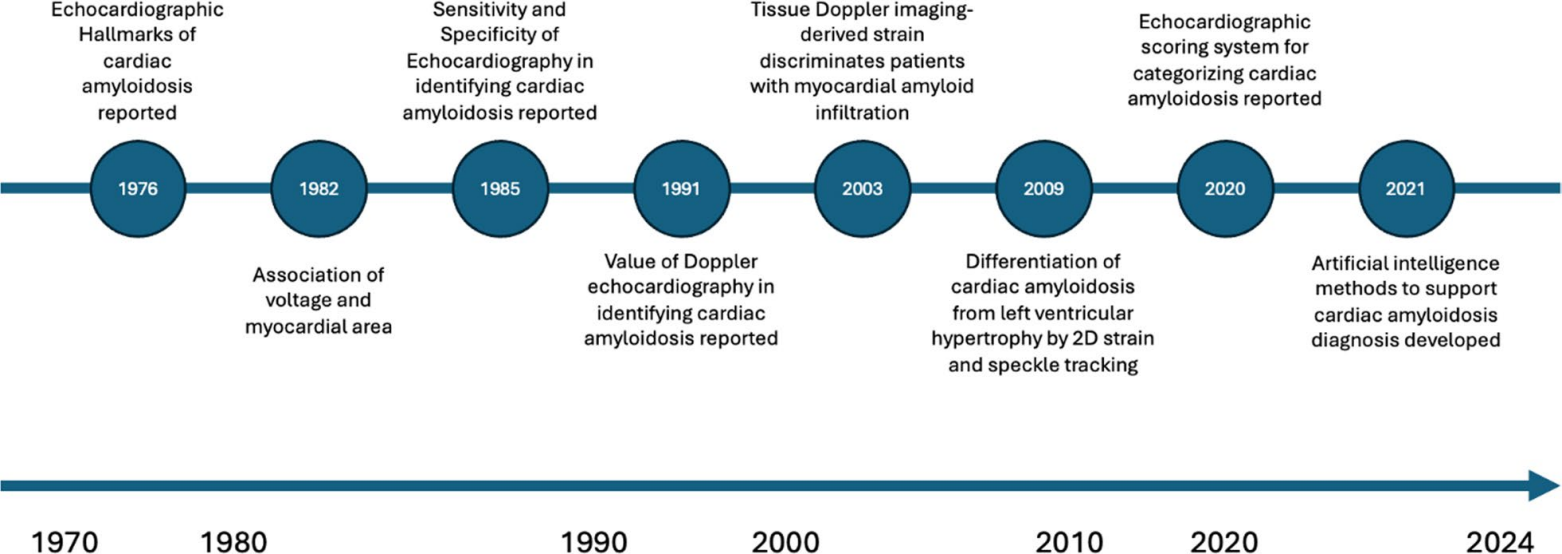
Timeline of echocardiography advancements in the identification and diagnosis of cardiac amyloidosis

**Fig. 4:**
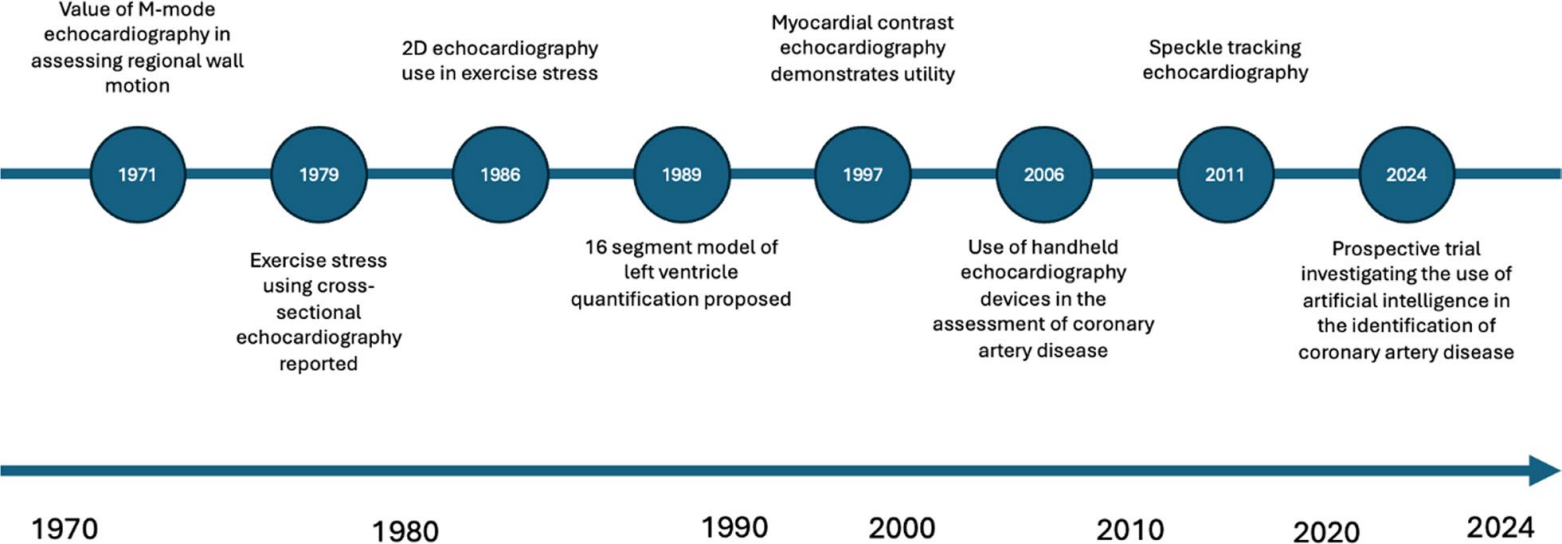
Timeline of echocardiography advancements in the identification and diagnosis of coronary artery disease

While much of echocardiography research began as small studies conducted by a few investigators at single institutions, advances in infrastructure and collaboration have allowed for the growth of larger-scale developmental studies [6]. This has enabled, echocardiography research to span from novel developments in technique refinement through small-scale experimental studies, to large-scale endeavours that have provided significant advances in clinical use [6, 20]. Technological advances through the 2000s have facilitated the development of collaborative research infrastructures, linking institutions internationally, and supported set up of larger datasets and cohorts. This has led to the development and integration of more advanced techniques such as 3D echocardiography and automated measurements [17] Figs. 2, 3, 4 illustrate three example disease areas, valvular heart disease [38–41], coronary artery disease [42–50], and cardiac amyloidosis [51–58] fundamental to echocardiography practice, in which disease identification and management has advanced over the life course of echocardiography, driven by research and development.

### Larger cohort studies and routinely collected data

As research methods for data capture and handling have become more accessible, opportunities for larger sample sizes have emerged, and so has the possibility of large-scale, population-based observational studies that utilise echocardiography. This has allowed exploration of the prognostic value of echocardiography and its role in clinical decision making. An overview of such studies and curated datasets is provided in Table 1 highlighting the advantages of each. The Framingham Heart Study was one of the earliest such studies assessing cardiac structure and function and included echocardiography measurements from the 1980s. This study provided early epidemiological data on left ventricular hypertrophy, identified echocardiographic predictors of atrial fibrillation, and demonstrated the prognostic value of left ventricular mass estimation by echocardiography [21–23]. Echocardiography also plays a large role in research of the Avon Longitudinal Study of Parents and Children, a prospective birth cohort of 14,000 mothers, and their offspring and partners in the UK, exploring how genetic and environmental factors affect cardiac structure, function, and development [24, 25]. The Atherosclerosis Risk in Community study [26], and the Multi-Ethnic Study of Atherosclerosis [27] are additional large-scale epidemiological studies focused on assessing factors that impact cardiovascular health across varied populations in the United States and has utilised echocardiography to investigate various cardiac diseases [28–30], examine the performance of echocardiography [31, 32] and establish reference values [33] particularly in populations underrepresented in clinical research. The World Alliance Societies of Echocardiography and the European Association of Cardiovascular Imaging conducted global observational studies to establish echocardiographic reference ranges in diverse populations [34–36].

**Table 1:** Illustration of large-scale epidemiological studies and databases in cardiovascular disease utilising echocardiography

Research study	Description	Advantages
Framingham Heart Study [21–23]	Landmark longitudinal US study started in 1948, key in identifying cardiovascular disease risk factors	Long-term follow-up, multi-generational, foundational epidemiological data in over 15,000 participants
Atherosclerosis Risk in Communities (ARIC) [26, 29, 30, 32]	Large US study exploring atherosclerosis risk factors in African American communities	Racial/ethnic diversity, robust cardiovascular phenotyping of participants often underrepresented in research
Multi-Ethnic Study of Atherosclerosis (MESA) [27, 28, 31, 33]	US study of subclinical atherosclerosis in diverse populations	Multi-ethnic cohort, high imaging standardisation, focus on subclinical disease and cardiac risk factors in over 6,000 participants
World Alliance Societies of Echocardiography (WASE) [34, 35]	Global collaboration assessing normal echo values across continents	Diverse population with international scope, cross-population normal reference values in over 2000 participants across 15 countries
EACVI NORRE (Normal Reference Ranges for Echocardiography) [36]	European initiative by EACVI to define normal values in healthy adults	Ethnic diversity with the inclusion of a uniform imaging protocol in over 700 healthy participants across 22 countries
Avon Longitudinal Study of Parents and Children (ALSPAC) [24, 25]	UK-based birth cohort study tracking health from childhood to adulthood	Lifespan data, intergenerational insights from over 14,000 pregnant women as well as offspring and grandchildren
EVAREST (Echocardiography Value at Rest and Stress) [37, 38]	Prospective national observational study to assess the performance of stress echocardiography in the UK	Illustrates current clinical practice in over 18,000 participants
NEDA (National Echo Database of Australia) [43, 44]	National registry linking echo data to long-term outcomes	Big data scale, real-world outcomes, population-wide analysis in over 600,000 participants
NED-UK (National Echocardiography Database—UK) [45–47]	UK equivalent to NEDA, large-scale echo registry linked to clinical records	Nationwide scale, will help establish prognostic value of echocardiography and curate datasets for AI model development
Stress Echo 2020 [40]	Prospective international study evaluating a multiparametric stress echocardiography protocol	Quality-controlled, global standardization of stress echo in over 3500 participants in five European countries
EchoNet-Dynamic [48, 49]	Large-scale echocardiogram dataset for use in AI model development	Over 10,000 echocardiogram images freely available as open-source dataset with continuous development for various cardiac diseases

In addition to epidemiological, population-based research there has been increasing interest in understanding real world applications of echocardiography. Recently, the EVAREST study assessed the performance of stress echocardiography in the identification of coronary artery disease in over 18,000 participants across 35 hospitals in the United Kingdom [37, 38], and has influenced recent guidelines in the management of chronic coronary syndromes [39]. The stress echocardiography 2020 study brought together numerous stress echocardiography laboratories across several countries to assess the prognostic value of a more comprehensive stress echocardiography protocol to address patient heterogeneity and emerging complexity of coronary artery disease [40]. The updated protocol demonstrated efficacy in predicting survival [41] and is now being further studied in the stress echocardiography 2030 expansion [42].

While there have been advances in large epidemiological, and population-based research studies in echocardiography, must insight can be gained from routine clinical echocardiograms. This data is gathered from real-world practice and thus is generalisable to the wider population, has a volume that is often unachievable and costly to acquire in research studies, and provides longitudinal data on disease progression and patient outcomes. The National Echo Database Australia (NEDA) has collected echocardiography data for over 600,000 patients [43], and is the largest routinely collected echocardiogram database in the world. The initiative has benchmarked ethical data acquisition, storage, and management practices while also advancing understanding of cardiovascular disease progression [44]. Such methodology is now being replicated in the UK through the National Echo Database UK (NED-UK) [45, 46]. Like NEDA, NED-UK looks to collect a large volume of echocardiographic data from electronic patient health records across the UK to help determine the diagnostic and prognostic value of echocardiography [47]. While NEDA and NED-UK only contain measurement and clinical report data, researchers at Stanford University have developed EchoNet-Dynamic, a large dataset of echocardiogram images complete with expert annotations, measurements, and calculations [48]. This dataset has helped support the development of deep learning models for the assessment of cardiac structure and function and is notably among the first datasets of this kind to be freely available as an open-source dataset [49].

### Randomised clinical trials

While observational studies are crucial to understanding effects of disease or treatment in ‘real-world’ settings, randomised clinical trials aim to evaluate new treatments or existing treatments for new use cases in tightly controlled conditions. Many randomised clinical trials and healthcare datasets benefit from a multi-centre design. These types of studies address limitations of research conducted at a single centre such as enhancing external validity and generalisability, increasing statistical power, all else being equal, and facilitating accelerated recruitment across numerous locations [50]. The scale of these studies has only been made possible through recent advances in IT infrastructure, enabling the secure collection, storage, and sharing of research data. Echocardiography is often an important component of randomised clinical trials and is often employed to visualise and quantify changes in cardiac structure and function in response to investigational pharmaceuticals and treatments.

Commonly, drug discovery trials employ the use of echocardiography as a monitoring and surveillance tool, particularly in oncology trials. The use of echocardiography at all stages in these randomised trials helps mitigate cancer therapy-related cardiac dysfunction and recognise early signs of potential cardiotoxicity. Routinely, left ventricular ejection fraction is widely used to evaluate patients during trials of cancer therapies, but conventional 2D methods may suffer from temporal variability, resulting in the cessation of treatment due to changes in left ventricular function only due to image acquisition variability. Ejection fraction with 3D techniques has shown a reduced temporal variability [51] and recent evidence also suggests that global longitudinal strain can detect subclinical disease and predict future left ventricular dysfunction [52]. With the current evidence, the use of left ventricular ejection fraction and global longitudinal strain have been proposed in the evaluation and management of cancer therapy-related cardiac dysfunction [53].

Echocardiography also plays a central role in cardiac disease drug development. These trials may include primary endpoints of cardiac-related mortality, hospitalisation, or composite endpoints of cardiac events as is the case for recent studies assessing medications for heart failure [54, 55] or functional response such as exercise capacity in trialling cardiomyopathy therapies [56, 57]. The inclusion of echocardiographic parameters as secondary endpoints provides further mechanistic insight of drug interaction through direct visualisation of cardiac structure and function. This can identify differential drug responses through cardiac phenotyping and identify surrogate imaging endpoints if carefully considered alongside risk of primary endpoint outcomes. While there is evidence supporting the utility of echocardiographic parameters to predict patient outcomes and support phase III clinical trials in drug development [58], other results have cautioned the overreliance of these measures due to observed discordant associations with primary endpoints [59].

To overcome limitations and produce more robust trial results, there is a need to optimise echocardiography protocols within clinical trials and select appropriate measures of cardiac structure and function to support the primary research objective. The American Society of Echocardiography have released recommendations highlighting appropriate uses of echocardiography in clinical trials, advocating for thorough training of site echocardiography operators with quality assurance practices in place, and the employment of an imaging core laboratory to provide consistent and reproducible results [60].

## Infrastructure for echocardiography research

**Fig. 5:**
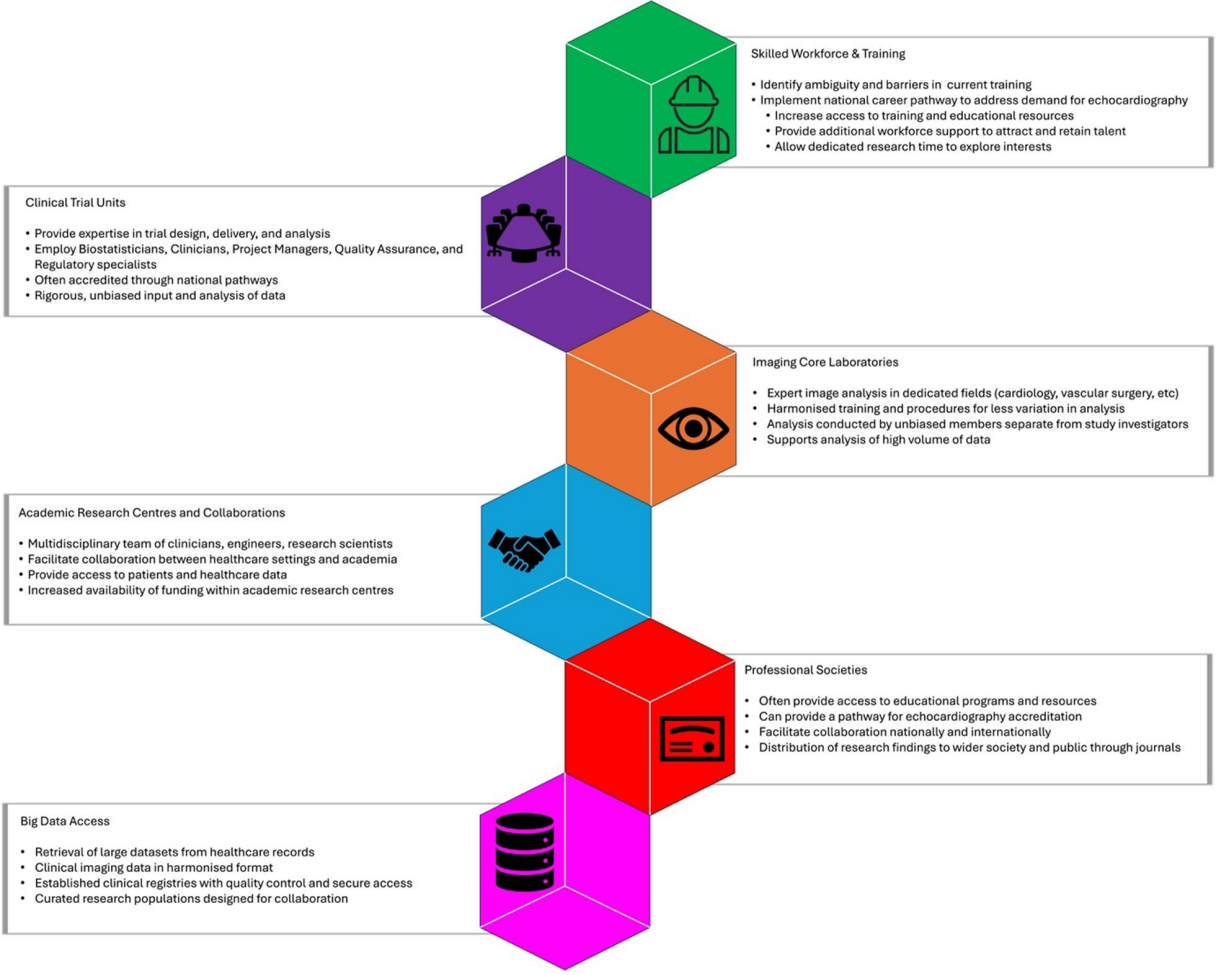
Six suggested infrastructure requirements to support echocardiography research

In the past few decades, echocardiography research has tended to develop, reactively, to support technology and clinical developments. There is an opportunity for a more strategic approach in the future, considering how research practice in echocardiography could be developed to support and accelerate future developments in clinical practice. Developments in echocardiography have been paralleled by growing interest in task force and consensus statements to guide clinical use of echocardiography across cardiac conditions such as valve disease [61], ischaemic heart disease [39], and heart failure [62]. Further, the European Association of Echocardiography have released suggestions for optimal echocardiography use in support of clinical trials [63]. The underlying guidance supporting these recommendations stems from crucial research efforts made possible only with the support of efficient research infrastructure and workforce. As a guide, several key components should be considered when establishing an ideal echocardiography research infrastructure, and Fig. 5 highlights suggested components to facilitate this.

### Skilled workforce and training

Clinical research can only exist with the support of healthcare staff and clinical expertise. Thus, supporting the echocardiography workforce is crucial to sustaining echocardiography research studies and trials. There is an ever-increasing demand for echocardiography workforce, but there are currently barriers and ambiguity in training and retention as identified by the British Society of Echocardiography [64]. The society has recently suggested a formal national career pathway in echocardiography which includes dedicated training, support, and research time. If implemented, this strategy has the potential to enhance the current workforce and promote research advancements. Embedding research and innovation into clinical practice and healthcare science is a stipulated requirement in UK department of health policy [65], and implemented in programmes such as the Specialist Training Programme [66] and the Higher Specialist Science Training Programme [67]. The inclusion of role models and mentorship, both academic and clinical, through programmes such as Mentor Match run by the American Society of Echocardiography [68], would ideally encourage participation and empower the workforce.

### Clinical trial units

Clinical trial units are specialised research centres that provide expertise in the design, delivery, and analysis of clinical trials and other research studies. These units often have experts in all aspects of the clinical research lifespan including biostatisticians, clinicians, project managers, quality assurance, and regulatory specialists. This expertise can supplement research groups who may otherwise lack experience in aspects of clinical research delivery. In the UK, clinical trial units must demonstrate excellence in the delivery of multi-centre clinical trials to gain UK Clinical Research Collaboration accreditation [69]. Further specialised centres can be sought if a particular trial requires dedicated expertise in a particular area such as cardiology or imaging. A survey of clinical trial units in the UK revealed a few key inefficiencies in clinical trial conduct that could be remedied with effective clinical trial unit support including data management and site training [70]. Recommendations included integrating site training across and improved communication between recruitment centres and study investigators. Image acquisition within echocardiography is often undertaken in accordance with national and international guidance. However, the employment of clinical trial units can support research sites with offering bespoke training in image protocols and data transfer and provide ongoing quality control which are all essential in gaining robust and complete echocardiography datasets.

### Imaging core laboratories

To overcome limitations of echocardiography, use such as variability in image acquisition and analysis, echocardiography imaging core laboratories are often employed, Inter-operator variability can impact the reliability of results [71], thus the importance of employing an imaging core lab with harmonised training and standard operating procedures cannot be understated. Imaging core laboratory analysts typically have years of experience in specific health areas such as cardiology or vascular surgery. This is crucially important in large scale, international clinical trials where local practices can vary greatly across participating hospitals. The ability of an external echocardiography core laboratory to reduce bias and maintain consistency in analysis has been previously demonstrated in the multi-centre ISCHEMIA trial [72]. Moreover, a post-hoc analysis of the PARTNER I trial in valvular heart disease revealed high quality and core lab reproducibility benefitting the trial when stringent protocols and quality assurance practices were introduced [73]. Furthermore, The American Society of Echocardiography has released standards for efficient echocardiography core laboratory practices, highlighting the integration of core labs within a trial in more aspects than just image analysis [63]. The importance of echocardiography core laboratory involvement extends to from early trial design, through to data analysis and final manuscript preparation and as such is crucial in the efficient deployment of clinical research.

### Academic research centres and collaborations

Academic research centres play a major role in the conduct of clinical research. These centres are often associated with healthcare institutions and have a proven track record of successful research endeavours supported by clinicians, engineers, and research scientists. These centres also typically have access to multimodality imaging infrastructure such as cardiac MRI, CT, and nuclear imaging to augment echocardiography research. The close collaboration with healthcare institutions provides access to large patient populations, and in countries with centralised health systems such as the National Healthcare System (NHS) in the UK, can further support multi-centre studies. Perhaps most importantly these centres are routinely provided with funding necessary to support intricate clinical research investigations There are numerous private and government bodies as well as charities to fund research projects in cardiac imaging and echocardiography. UK Research and Innovation cites medical imaging as an area of investment and support [74], and supports the Medical Research Council which funds all forms of research to improve patient health. The British Heart Foundation is the largest independent funder of heart and circulatory disease research in the UK and will contribute £1bn in research over the next ten years [75]. Importantly, the foundation places a large emphasis on cardiovascular imaging as evidenced by their over 250 currently funded projects in imaging [76]. The National Institute for Health and Social Care Research have provided funding to establish 20 Biomedical Research Centres across the UK. These centres facilitate collaboration between local hospitals and academic centres and includes imaging as one of the key themes for research [77]. Within cardiology the NIHR-BHF Partnership has provided a means to develop research proposals collaboratively within key priority disease areas that typically require involvement of echocardiography expertise.

### Professional societies

**Fig. 6:**
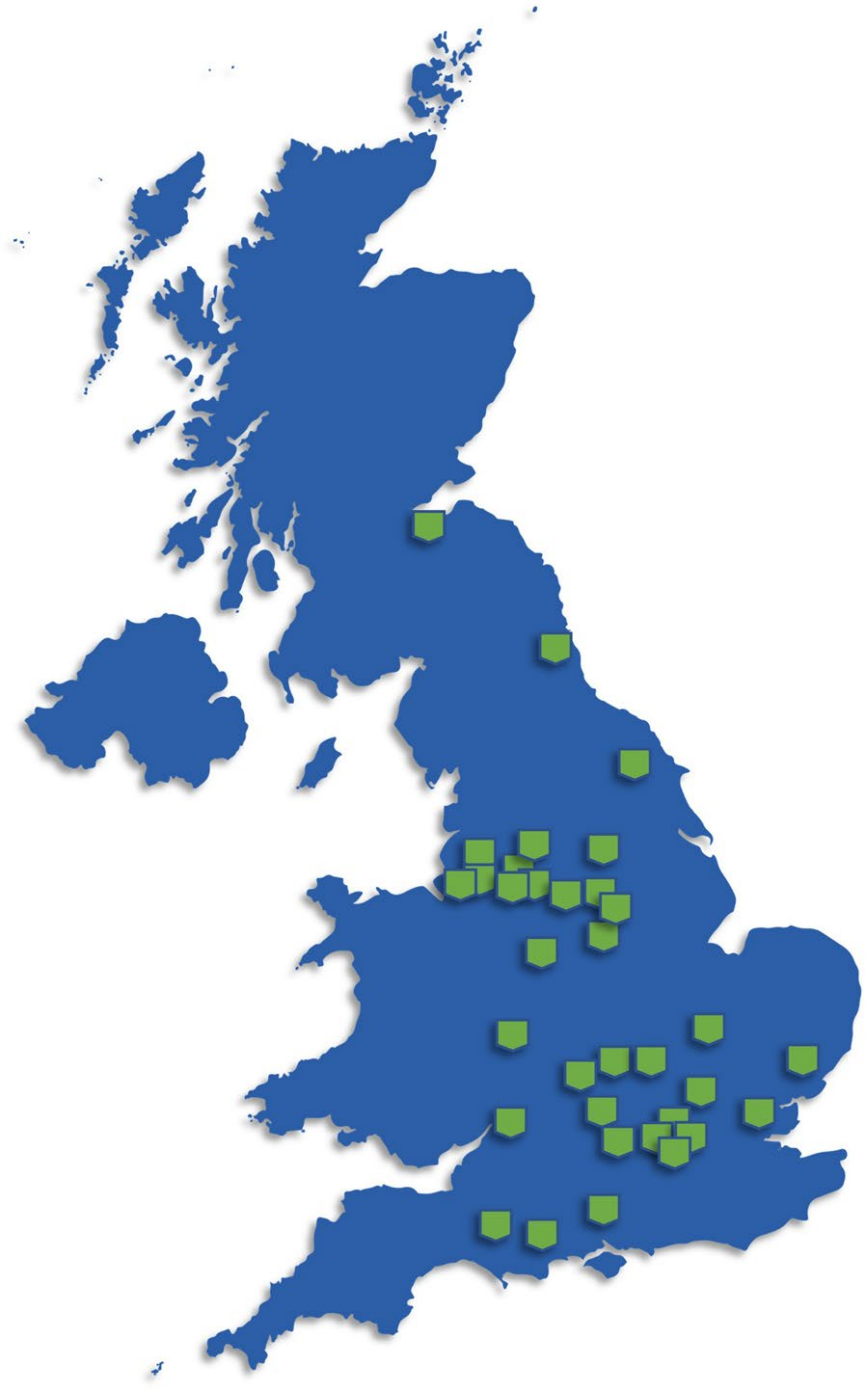
The British Society of Echocardiography established centres in the Echocardiography Research Network

Globally, societies exist to not only establish guidelines for echocardiography use but also facilitate research collaboration. The European Association of Cardiovascular Imaging encompasses numerous European societies focused at supporting echocardiography professionals in both clinical and research environments. Similar societies exist in the Americas, Asia–Pacific, the Middle East, and Africa. Perhaps most importantly, the UK benefits from The British Society of Echocardiography—an organisation aimed at providing not only training, accreditation, and career development, but supporting advancements in echocardiography through research in partnership with the British Cardiovascular Society and the British Heart Foundation. The society has also conducted national workforce surveys to better ascertain the current state of echocardiography capacity, demand, and education [78]. A recent development from the British Society of Echocardiography has been the establishment of a national echo research network (Fig. 6). The network has been designed to assist researchers and research teams connect and exchange ideas while supporting each other from study design through to delivery. Many of these societies maintain peer-reviewed journals and publish high quality clinical and basic research, reviews, guidelines and educational materials such as The British Society of Echocardiography’s *Echo Research and Practice*, or the *Journal of the American Society of Echocardiography*. The establishment of annual conferences further encourages collaboration and discussion amongst the community of members. All this supports the research and development of new cardiac ultrasound methods and technologies aiming to improve patient health and care.

### Big data access

Advancements in technology infrastructure has facilitated the era of ‘big data’ whereby large, diverse amounts of data are being collected from various sources. Key sources of data are electronic patient health records, medical imaging repositories, clinical trial registries, and laboratories to name a few [79]. The use of big data is driving innovations by applying AI and machine learning methods, allowing for improved predictive models, risk stratification, and precision medicine. Suggestions for echocardiography in the age of big data have been proposed [80], highlighting the potential big data has to revolutionise echocardiography research and practice with the appropriate data science skills and collaborations with expert data scientists. Centralised healthcare systems such as the NHS in the UK are well-equipped to provide large amounts of patient data to research teams through established access platforms. Currently new NHS Secure Data Environments are being developed within NHS England, with similar structure existing in the NHS within the other UK devolved administrations. These will provide a means for much larger scale analysis of routinely collected NHS data, including echocardiography, for research purposes.

The British Society of Echocardiography is contributing to the available infrastructure to support pragmatic clinical research by funding early stages of the new National Echocardiographic Database of the UK [81]. NED-UK aims to provide a mechanism of extraction and storage of anonymised echocardiographic reports which can be linked to patient outcomes. This will create a national database to support future research projects at scale. The preliminary work of NED-UK has recently been presented [45, 46], and ongoing work continues to investigate the linkage of this work with newly introduced secure data environments established by NHS England [82].

Clinical registries exist in a wide variety of health conditions and are rich data sources that collect data from clinical settings, often with a longitudinal design allowing for assessment of patient outcomes long-term [79]. Outside of the healthcare setting, there exist curated research populations consisting of hundreds of thousands of patient records such as the UK Biobank [83], the German National Cohort [83], and the Atherosclerosis Risk in Communities Study [84].

## Future opportunities for echocardiography studies

**Fig. 7:**
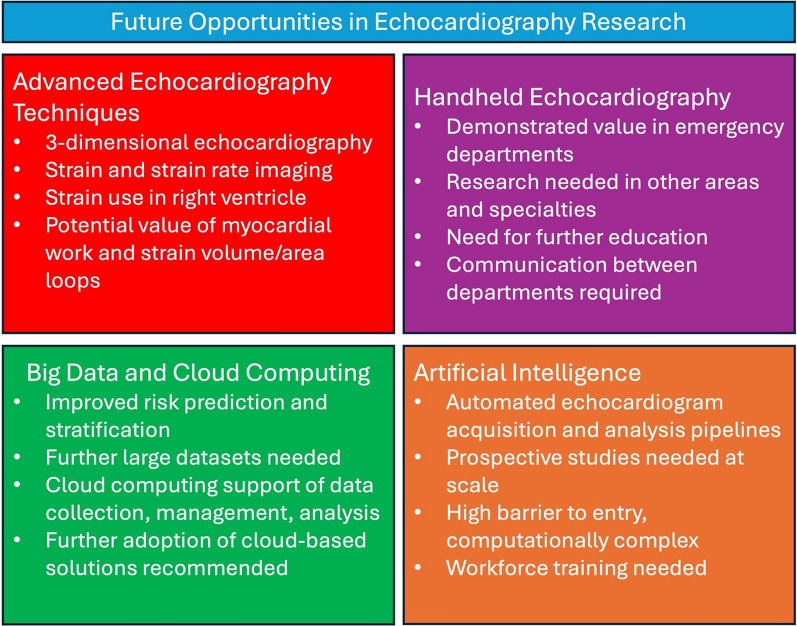
Key future opportunities in the field of echocardiography research and practice

The field of echocardiography research and clinical practice is highly promising, driven by technological advancements provided through numerous research efforts. While a great amount of work has been conducted to date, there are numerous technologies that require continuous research and development to ascertain how patients can best benefit as shown in Fig. 7.

### Advanced echocardiography techniques

Standard echocardiography has proven instrumental in the assessment of cardiac function, and recent developments in advanced echocardiography techniques such as 3D echocardiography and myocardial strain imaging have demonstrated promising clinical implications [85, 86]. Previous reviews have suggested that increased implementation of 3D echocardiography will improve the clinical workflow and patient outcomes but requires further rollout to more than just tertiary centres and requires further research into cost-effectiveness to inform guidelines and policy [87, 88]. There is also a need to improve the standardisation of 3D echocardiography which has remained a barrier to widespread clinical implementation [89]. Automated 3D techniques have demonstrated promise at producing reliable and reproducible measurements across varying centres [89], but further work is warranted, especially considering variations in techniques across echocardiography vendors. The prognostic utility of 3D echocardiography has been demonstrated [90, 91] and is of particular of value in the assessment of right ventricular structure and function which can be difficult to evaluate using traditional 2D techniques [35]. However, there are technical limitations to 3D echocardiography use which require addressing such as reduced frame rate, and difficulties when combined with contrast enhancement, compromising spatial and temporal resolution [92] Additional developmental work is needed to overcome these limitations and increase adoption of 3D echocardiography into clinical practice.

The development of speckle tracking and its use in myocardial strain imaging has allowed for the quantification of ventricular function beyond that of subjective image interpretation [86]. Strain imaging provides a more comprehensive analysis of myocardial function through the assessment of shortening or lengthening within the myocardium [85]. Global longitudinal strain has been shown to be a predictor of all-cause mortality and provides further diagnostic information beyond left ventricular function alone in mild systolic function [93]. While prior research in strain imaging has led to its adoption in clinical practice, there are still challenges warranting further investigation. Some studies have demonstrated the value of strain imaging in the right heart assessment [94–96], and 3D echocardiography appears to further support this [35], but additional evidence is needed. The use of left atrial strain has been included in recent guidelines for the assessment of diastolic dysfunction [97], and future work validating this guideline update against patient management and outcomes will be needed. The use of strain rate may be superior to peak systolic strain as it has been shown to be less load dependent [98], but requires high frame rates [99]. Further research in ultra-high frame rate echocardiography may mitigate this limitation [100, 101]. Lastly, there is a growing interest in strain volume/area loops in the assessment of myocardial work which can quantify the relationship between strain and volume across the cardiac cycle, but the diagnostic and prognostic value of this technique remains unclear and advocates of this technique have highlighted the need for further studies to evaluate prognostic value [102, 103].

### Handheld echocardiography

The development and use of handheld echocardiography devices has become an area of interest in recent years with the major ultrasound vendors providing solutions such as the Philips Lumify™ and GE Vscan™ devices. These devices offer portability, affordability, and are an easier introduction to echocardiography scanning. The use of portable echocardiography solutions is routinely discussed alongside focused cardiac ultrasound assessment and have demonstrated comparable results to standard on-cart imaging [104, 105]. Interest in the use of focused cardiac assessment in the emergency setting has increased in recent years, and as part of a multiorgan assessment, it has been demonstrated that rapid cardiac ultrasound can effectively identify patients with heart failure [106, 107], cardiac tamponade, [108] or significant valve disease [109].

While the use of handheld echocardiography devices as part of a focused cardiac assessment has been demonstrated mostly in emergency settings, there is a lack of research assessing their use in other settings such as primary care and family medicine [110]. It is expected that the number of non-cardiology specialists performing focused cardiac assessments by echocardiography will increase [111], and there are concerns regarding the performance of echocardiography in the hands of nonspecialists [110, 112]. Further evidence surrounding educational support, training and governance for handheld echocardiography practice is welcome.

### Big data and cloud computing

Big data is already in use in everyday life when following directions on navigation applications, or when faced with personalised ads online. The application of big data in a healthcare context is still in its infancy, but there are several potential applications of big data analytics such as risk prediction modelling, quality assessment of healthcare, and precision medicine [113]. Having access to a large, anonymised dataset of patients undergoing echocardiography would have similar applications and is becoming increasingly feasible as technology advances.

One of the most common applications of such datasets is the development of prediction models using deep learning or AI methods. These methods aim to identify high-risk patients who may benefit from closer observation or more personalised management [113]. Similar methods have already been investigated using multi-modality imaging [114], but lack echocardiographic data. Employing AI assessment of big data in risk stratification looks to overcome limitations of current methods of identifying risk by incorporating more covariates and input features than previously possible [115]. The establishment of dedicated echocardiography databases such as NEDA and NED-UK will prove crucial in facilitating further big data research in echocardiography.

Increasingly, health data is being stored, accessed, and analysed in cloud services [116]. While big data is concerned with collecting and handling data, cloud computing is required to provide a method to manipulate, process, and analyse the data that has been acquired. There benefits of cloud computing to the field of echocardiography research and practice are vast. These methods look to support faster diagnoses and reduce waiting times, and with the support of AI, reduce human error and variability, and provide information nearly instantaneously from a variety of sources. Not only are there benefits to improving patient outcomes, but such infrastructure can significantly reduce costs and maintain high levels of security [117]. Such examples already exist in echocardiography such as Ultromics Ltd. [117], and US2.ai [118], medical technology companies offering cloud-based AI echocardiogram analysis. An increased awareness of such cloud-based services is crucial in successful implementation into research and clinical workflows.

### Artificial intelligence

The use of AI has received widespread attention in the past decade and has been increasingly investigated in medical imaging. AI and its complement big data have already made contributions to echocardiography, from nuanced image acquisition and quality control features pre-built into ultrasound machines, to third party diagnostic decision support algorithms and software. Several reviews have assessed the field of AI in echocardiography [119–122] highlighting notable developments and future suggestions. Commercial systems already include solutions in border delineation, ejection fraction and global longitudinal strain analysis [123–128]. Some fully automated pipelines have also been developed, utilising AI across all aspects of echocardiography from view identification and image segmentation to full analysis and disease detection [129, 130]. Recent research has demonstrated the utility of AI-guided image acquisition, potentially increasing echocardiography adoption and accuracy in the hands of novices and non-specialists [131, 132].

Despite these advances, the need for prospective real-world testing of AI technologies is an important next step in the roadmap to AI implementation into clinical practice [133], but few models have been tested prospectively [134]. He et al. [135] recently tested the automated assessment of ejection fraction in a blinded, and randomised fashion, adding substantial evidence in favour of AI support in cardiac assessment. Fewer studies still have tested AI technologies in an interventional manner prospectively and assessed impact on patient outcomes. The PROTEUS trial [136, 137] evaluated the use of AI in detecting severe coronary artery disease by analysing stress echocardiography images. The successful deployment of the trial is encouraging and will hopefully usher in further studies focused on the implementation of AI technologies into clinical practice.

The potential applications of AI to echocardiography research and practice are vast, but there are still several research requirements that need to be addressed to best facilitate further development and fill the current gaps in research. The development of AI solutions requires high quality, diverse data at scale [138]. While there are a few initiatives and collaborations looking to curate such datasets [44–46, 48], additional efforts are required in this area. Researching using AI methodologies is also technologically and computationally complex often requiring specialised expertise, and computing infrastructure [138], which can pose significant barriers to entry. This further highlights the importance of multidisciplinary collaboration across both academic and clinical centres of all experience levels. Such collaboration is a key component in addressing the paucity of real-world testing of AI, and the establishment of research networks is a step forward in preparation of the many future endeavours in the investigation of AI in echocardiography research and practice.

## Conclusion

While substantial strides have been made in the field of echocardiography research and practice, numerous opportunities remain to deepen our understanding of this technique and further optimise its use to improve patient outcomes. In this article, we have highlighted the contributions of echocardiography research to date, distinguishing the insights gained from early small-scale observational studies at single centres from those of large cohort studies and randomised controlled trials. We have explored the multifaceted role of echocardiography in clinical trial endpoints and discussed how advancements in disease identification and diagnosis have paralleled the development of echocardiographic techniques. Additionally, we have outlined key infrastructure requirements needed to foster collaboration and support the continuing evolution of echocardiography in the context of the fast-moving digital age. In view of the infrastructure already in place the UK is well equipped to be a leader in echocardiography research. Looking ahead, advanced imaging techniques, the integration of big data, and the adoption of AI hold the potential to overcome current limitations in clinical practice. These advancements promise to revolutionise the field and drive improvements in patient outcomes on a global scale.

## Data Availability

No datasets were generated or analysed during the current study.

## Abbreviations

2D: 2-Dimensional
3D: 3-Dimensional
AI: Artificial Intelligence
NEDA: National Echo Database Australia
NED-UK: National Echo Database UK
NHS: National Healthcare System
